# Correlating the electronic structures of metallic/semiconducting MoTe_2_ interface to its atomic structures

**DOI:** 10.1093/nsr/nwaa087

**Published:** 2020-04-29

**Authors:** Bo Han, Chen Yang, Xiaolong Xu, Yuehui Li, Ruochen Shi, Kaihui Liu, Haicheng Wang, Yu Ye, Jing Lu, Dapeng Yu, Peng Gao

**Affiliations:** Electron Microscopy Laboratory and International Center for Quantum Materials, School of Physics, Peking University, Beijing 100871, China; Department of Material Physics and Chemistry, University of Science and Technology Beijing, Beijing 100083, China; State Key Laboratory for Artificial Microstructure & Mesoscopic Physics, School of Physics, Peking University, Beijing 100871, China; Academy for Advanced Interdisciplinary Studies, Peking University, Beijing 100871, China; State Key Laboratory for Artificial Microstructure & Mesoscopic Physics, School of Physics, Peking University, Beijing 100871, China; Electron Microscopy Laboratory and International Center for Quantum Materials, School of Physics, Peking University, Beijing 100871, China; Electron Microscopy Laboratory and International Center for Quantum Materials, School of Physics, Peking University, Beijing 100871, China; State Key Laboratory for Artificial Microstructure & Mesoscopic Physics, School of Physics, Peking University, Beijing 100871, China; Collaborative Innovation Center of Quantum Matter, Beijing 100871, China; State Key Laboratory of Advanced Materials for Smart Sensing, GRINM Group Co. Ltd., Beijing, and GRIMAT Engineering Institute Co. Ltd., Beijing 101402, China; State Key Laboratory for Artificial Microstructure & Mesoscopic Physics, School of Physics, Peking University, Beijing 100871, China; Collaborative Innovation Center of Quantum Matter, Beijing 100871, China; State Key Laboratory for Artificial Microstructure & Mesoscopic Physics, School of Physics, Peking University, Beijing 100871, China; Collaborative Innovation Center of Quantum Matter, Beijing 100871, China; Shenzhen Key Laboratory of Quantum Science and Engineering, Shenzhen 518055, China; Electron Microscopy Laboratory and International Center for Quantum Materials, School of Physics, Peking University, Beijing 100871, China; Collaborative Innovation Center of Quantum Matter, Beijing 100871, China

**Keywords:** MoTe_2_, phase engineering, coplanar phase boundary, EELS

## Abstract

Contact interface properties are important in determining the performances of devices that are based on atomically thin two-dimensional (2D) materials, especially for those with short channels. Understanding the contact interface is therefore important to design better devices. Herein, we use scanning transmission electron microscopy, electron energy loss spectroscopy, and first-principles calculations to reveal the electronic structures within the metallic (1T^′^)-semiconducting (2H) MoTe_2_ coplanar phase boundary across a wide spectral range and correlate its properties to atomic structures. We find that the 2H-MoTe_2_ excitonic peaks cross the phase boundary into the 1T^′^ phase within a range of approximately 150 nm. The 1T^′^-MoTe_2_ crystal field can penetrate the boundary and extend into the 2H phase by approximately two unit-cells. The plasmonic oscillations exhibit strong angle dependence, that is a red-shift of π+σ (approximately 0.3–1.2 eV) occurs within 4 nm at 1T^′^/2H-MoTe_2_ boundaries with large tilt angles, but there is no shift at zero-tilted boundaries. These atomic-scale measurements reveal the structure–property relationships of the 1T^′^/2H-MoTe_2_ boundary, providing useful information for phase boundary engineering and device development based on 2D materials.

## Teaser

The properties of 1T^′^/2H-MoTe_2_ phase boundaries are measured at atomic-scale and found to be angle-dependent, providing guidelines to tune the local band structure and contact resistance.

## INTRODUCTION

Two-dimensional (2D) transition metal dichalcogenides (TMDs) have attracted extensive attention for their potential applications in nanoelectronics [1,2]. In atomically thin TMD devices, contact interface properties can significantly influence the performance, particularly in short-channel devices [3,4]. An imperfect interface between the electrode and a 2D semiconducting TMD can cause Fermi level pinning and thus result in high resistance across the contact [2,5], which limits potential applications as device sizes scale down. Recent strategies such as indium/gold contacts [6], tunneling contacts [7], and metallic 2D material contacts [8] have been used to reduce contact resistance in long-channel devices [3,6–9]. However, these techniques are less effective in short-channel devices or large-scale applications. Recently, heterophase (e.g. metallic 1T^′^-MoTe_2_ [10,11] and semiconducting 2H-MoTe_2_ [12,13]) coplanar structure [14–16] have been demonstrated to effectively reduce contact resistances in stable integrated circuits [17] by avoiding introduction of defects and impurities from step-by-step device fabrication processes [18–21]. These keep the promise of phase engineering as an effective way to reduce short-channel device contact resistances to achieve the low contact resistance requirements of the International Technology Roadmap for Semiconductors [4].

The properties of these coplanar boundaries (e.g. 1T^′^/2H-MoTe_2_) should be dictated by their atomic structures, such as the interfacial sharpness, relative orientation between metallic and semiconducting phases, and nature of the interfacial bonds, which, unfortunately, remain largely unknown because of a lack of techniques that correlate the electronic structures of atomically thin interfaces to their microstructures. Conventional optical measurements generally offer neither sufficient spatial resolution to probe the local properties of interfaces and defects, nor the ability to determine their atomic structures. Scanning tunneling microscopy/spectroscopy (STM/STS) techniques are typically sensitive only to the energy density of states (DOS) near the Fermi level with respect to TMD interfaces [22–25]. As a result, the dependence of deep ultraviolet (DUV) range plasmonic properties and inner shell transitions on atomic structure has rarely been investigated with high spatial resolution. Recent advances in electron energy loss spectroscopy (EELS) in scanning transmission electron microscopy (STEM) with monochromator allow for probing of the inter-band transition [26–29] and even lattice vibration [30–32], including thermometry [33], isotopic labeling [34], and phonon dispersion diagram measurement [35], at high spatial resolution. Therefore, EELS in electron microscopes with a probe corrector and monochromator offers a solution to correlate the electronic properties to atomic structures of coplanar boundaries.

Here, we use monochromatic STEM-EELS with sub-10 meV energy and atomic spatial resolutions to study 1T^′^/2H-MoTe_2_ phase boundaries. We correlate the atomic structure of each phase boundary with its electronic states over a wide spectral range from hundreds of meV to hundreds of eV. We find that the interband transition behavior of MoTe_2_ exhibits delocalized character within approximately 150 nm at all 1T^′^/2H phase boundaries with various tilt angles (relative orientations). The DUV plasmon oscillation (π+σ) peak has a red-shift of approximately 0.3–1.2 eV within 4 nm of the boundary at large tilt angles as a result of a change in the dielectric function and decreased free electron density. No substantial shift is observed for those boundaries with small tilt angles, which indicates that the relative orientations of the two crystal grains have significant influence on the contact properties. Furthermore, the interactions between 1T^′^ and 2H phases change the crystal fields at all phase boundaries and thus alter the energy-loss near-edge structures (ELNES) of the Te-N and Te-M edges within approximately two unit-cells of the boundary on the 2H-MoTe_2_ side. These findings of microstructure-dependent electronic structures at 1T^′^/2H-MoTe_2_ phase boundaries could help us to understand device contact properties and further guide design of high-performance nanodevices via coplanar boundary engineering.

## RESULTS AND DISCUSSION

**Figure 1.**
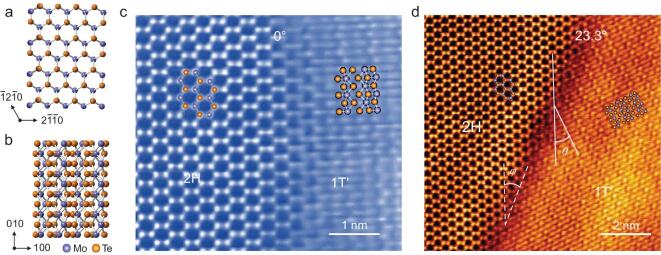
Atomic structures of 1T^′^/2H-MoTe_2_ coplanar boundary. Atomistic models of (a) 2H-MoTe_2_ and (b) 1T^′^-MoTe_2_ viewed from [001]. (c) An atomically resolved HAADF image of a MoTe_2_ metallic (1T^′^)/semiconducting (2H) coplanar boundary with a tilt angle of ∼0°. (d) A HAADF image of ∼23.3° tilted 1T^′^/2H boundary. The tilt angle *θ* of the MoTe_2_ boundary is determined by the zigzag direction of the 2H phase and the [010] direction of the 1T^′^ phase (white solid lines).

As depicted in Fig. 1a and b, the atomic structures of the 2H-MoTe_2_ and 1T^′^-MoTe_2_ phases are remarkably distinct. Unlike 2H-MoTe_2_, in which the Mo and Te atoms have a regular prismatic arrangement, 1T^′^-MoTe_2_ exhibits a distorted atomistic arrangement, that is one Te-atom layer is offset from the next, resulting in octahedral coordination structures arranged around Mo atoms. The distinct atomic arrangements of the interlayer of 1T^′^ and 2H are shown in Fig. S1 in the online supplementary data. The connection between these 1T^′^ and 2H phases leads to formation of phase boundaries with different geometries, as illustrated in the high-angle annular dark-field (HAADF) images in Fig. 1c and d. Each 1T^′^/2H phase boundary has two angle parameters (as marked in Fig. 1d): the boundary rotation angle *ϕ* between the zigzag (<11$\bar{2}$0>) direction of the 2H phase and the boundary plane/line, the other is the tilt angle *θ* between the zigzag of the 2H phase and the [010] of the 1T^′^ phase. We label only the latter parameter *θ*, as the acquired data show no clear dependence on *ϕ*. To improve measurement accuracy, tilt angles are determined in reciprocal space (see details in Fig. S2 in the online supplementary data). The measurement uncertainties are discussed in detail in Fig. S3 in the online supplementary data.

**Figure 2.**
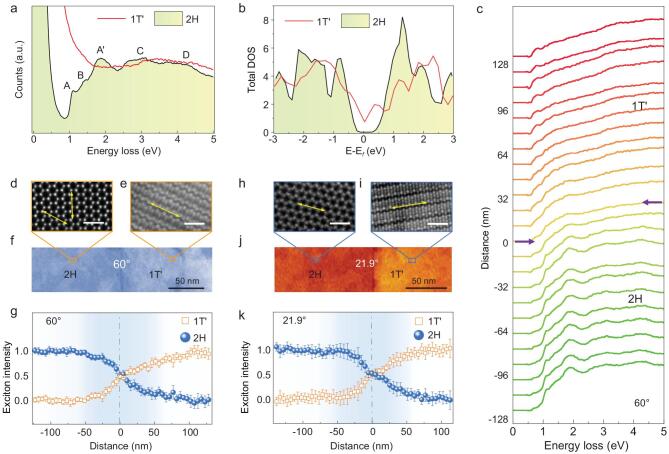
VEELS of 1T^′^/2H-MoTe_2_ phase boundaries. (a) VEELS of the intrinsic 2H- (black line) and 1T^′^-MoTe_2_ (red line). (b) Calculated DOS of 2H-MoTe_2_ (black line) and 1T^′^-MoTe_2_ (red line). Unit of vertical coordinates: states/(eV. atom). 2H-MoTe_2_ shows an intrinsic bandgap. (c) The VEELS series across a ∼60° tilted 1T^′^/2H phase boundary. The purple arrows indicate the location of the 1T^′^/2H-MoTe_2_ phase boundary. Magnified atomically resolved HAADF images of (d) 2H-MoTe_2_ and (e) 1T^′^-MoTe_2_ are used to determine angle of (f) the ∼60° tilted phase boundary. Scale bar: 1 nm. The HAADF images of the ∼60° tilted boundary show the corresponding spectral collection region (51 × 256 nm). (g) The exciton intensity versus distance across the ∼60° 1T^′^/2H-MoTe_2_ boundary. Atomically resolved HAADF images of (h) 2H-MoTe_2_ and (i) 1T^′^-MoTe_2_ are used to determine the angle of (j) a ∼21.9° tilted phase boundary. Scale bar: 1 nm. (k) The exciton intensity as a function of distance across the ∼21.9° 1T^′^/2H-MoTe_2_ boundary. Blue spheres: 2H; orange squares: 1T^′^. The gray dashed lines label the locations of the phase boundaries.

The valence electron energy-loss spectra (VEELS) shown in Fig. 2a demonstrate different valence electron transitions between 2H-MoTe_2_ and 1T^′^-MoTe_2_. The VEELS of 2H-MoTe_2_ contain five exciton peaks that represent its interband transitions [36] (see details in Fig. S4 in the online supplementary data). Unlike 2H-MoTe_2_, the spectrum of 1T^′^-MoTe_2_ comprises only one broad peak. This is caused by the absence of an energy gap near the Fermi level, as per the calculated DOS shown in Fig. 2b. The bandgap of semiconducting 2H-MoTe_2_ is 0.9 eV (see Fig. S4 in the online supplementary data), which is consistent with previous optical

measurements [10,36,37]. Figure 2c shows that a series of evanescent peaks extends across the phase boundary from 2H-MoTe_2_ to 1T^′^-MoTe_2_ (∼60° or 0° in tilt). This behavior likely stems from the long-range Coulomb interactions between incident beam and excitons, that is the swift electron-induced electromagnetic fields can excite the MoTe_2_ to generate electron-hole pairs even when the electron probe is distanced tens of nanometers away from the sample [28]. The tilt angle of ∼60° phase boundary is determined by the atomically resolved HAADF images in Fig. 2d and e. The VEELS are acquired within a 51 × 256 nm region containing the ∼60° tilted boundary, as shown in Fig. 2f. The interaction range of the ∼60° tilted phase boundary, from Fig. 2g, is fitted to be approximately 150 nm around the phase boundary (the fitting strategy is included in the Method section of the online supplementary data). Measurements from other phase boundaries with different tilt angles show that the typical interaction width is approximately 100–150 nm and there is no distinguished angle dependence (Fig. 2h–k; Fig. S5 in the online supplementary data).

**Figure 3.**
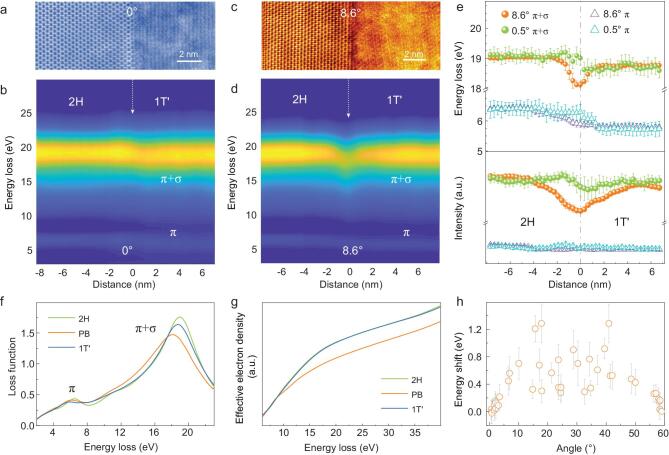
Plasmon oscillations at 1T^′^/2H-MoTe_2_ phase boundaries. (a) A HAADF image showing the spectral collection region (6 × 16 nm) containing a boundary with a tilt angle of ∼0°. (b) The spatially resolved plasmon oscillation of the ∼0° 1T^′^/2H-MoTe_2_ boundary. The white arrow indicates the location of the phase boundary. The π-mode energy loss peaks of MoTe_2_ are located at 6.4 eV (2H) and 5.7 eV (1T^′^). The energy value of the π+σ mode peak is ∼19 eV. (c) A HAADF image of a phase boundary with a tilt angle of ∼8.6°. (d) The corresponding plasmon spectra of the ∼8.6° tilted phase boundary. (e) Energy loss values and intensities of plasmon resonance peaks versus distance across the phase boundary. The phase boundary is indicated by the gray dashed line and determined from the corresponding HAADF image. (f) The loss functions of MoTe_2_. (g) The effective electron densities of MoTe_2_ (green: 2H-MoTe_2_; blue: 1T^′^-MoTe_2_; orange: at the phase boundary). (h) The energy red-shift of π+σ mode at phase boundaries with various tilt angles. The error bars indicate standard deviations calculated from positions within 0.8 nm of the phase boundaries.

STEM-EELS has the ability to probe plasmon oscillations in the DUV range (typically higher than 5 eV) with ultra-high spatial resolution. Figure 3a and b shows plasmon modes collected from a 6 nm × 16 nm area that contains a ∼0° MoTe_2_ phase boundary. Two dominant peaks π and π+σ can be observed in the 5–35 eV energy loss range [38], which is consistent with the theoretical calculations in Fig. S6 in the online supplementary data. In contrast to the small energy shift of the ∼0° phase boundary, the energy shift and intensity change at the ∼8.6° phase boundary are substantial (Fig. 3c and d). From the extracted energy loss values and intensities of the DUV plasmon oscillation modes shown in Fig. 3e, the

energy loss of π+σ peaks dramatically decrease about 0.75 eV within 4 nm at the ∼8.6° phase boundary. The peak intensity also decreases within the ∼8 nm region. This is likely because of defective bonds at the phase boundary.

The energy loss of the π+σ plasmon oscillation peak ${E_p}$ is determined by

$$\begin{equation*}
{E\!_p} = {h} \sqrt {\frac{{n{e^2}}}{{{\varepsilon _0}m}}},
\end{equation*}$$

where *n* represents the density of free charges, *e* is the electron charge, ${\varepsilon _0}$ is the permittivity of free space, and $m$ represents the effective electron mass. The red-shift of the π+σ plasmon mode at the phase boundary is attributed to a reduction in the effective electron density at the phase boundary. This is consistent with the Kramers-Kronig (K-K) analysis in Fig. 3f and g, as well as Fig. S7 in the online supplementary data. To identify the angle-dependent electronic properties of MoTe_2_ phase boundaries, we investigated various tilt angles (Fig. 3h). At boundaries with large tilt angles, the significant red-shift of the π+σ plasmon mode (19 eV) indicates weaker σ bonds (i.e. weak interactions between the σ electron clouds of Mo and Te atoms), which harm carrier injection. The subtle energy shift of the π+σ mode at the near zero-tilted phase boundary avoids this high carrier injection barrier. In this sense, the phase boundary tilt angle can be used as a knob to tune contact properties of coplanar structure.

**Figure 4.**
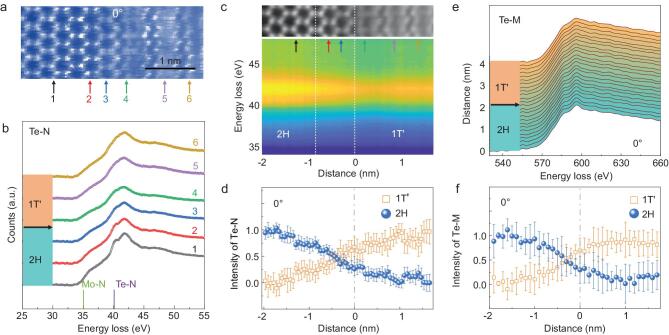
ELNES at the 1T^′^/2H-MoTe_2_ boundary. (a) A HAADF image showing the spectral collection region. (b) Te-N ELNES at six locations in the vicinity of the phase boundary are indicated by black, red, blue, green, purple and yellow curves, respectively. (c) A Te-N ELNES intensity map at the 1T^′^/2H boundary. The Te-N edge of 2H-MoTe_2_ changes rapidly within approximately two unit-cells near the boundary (highlighted by white dashed lines). (d) The Te-N ELNES intensities of 1T^′^ and 2H components across the boundary. (e) The Te-M ELNES across the 1T^′^/2H boundary. (f) The Te-M ELNES intensities of 1T^′^ and 2H components as a function of the distance across the boundary. The gray dashed lines indicate the locations of the 1T^′^/2H-MoTe_2_ phase boundaries.

The inner shell electronic structure of the 1T^′^/2H-MoTe_2_ phase boundary is also studied. The atomically resolved STEM-EELS in Fig. 4a and b illustrate that the Te-N edge of 2H-MoTe_2_ contains two peaks at 40.5 eV and 42 eV, whereas they are not well separated in the 1T^′^ phase. Similarly, the Mo-N edge is more pronounced in 2H-MoTe_2_ than that in 1T^′^-MoTe_2_. From the EELS intensity map in Fig. 4c, at various positions on both sides of the phase boundary, the Te-N edge of 2H-MoTe_2_ is altered only two unit-cells away from the phase boundary plane. The variation of 2H and 1T^′^ components shown in Fig. 4d also confirms that the Te-N ELNES of 2H-MoTe_2_ deviates from the intrinsic shape near the phase boundary. This subtle change in the Te-N edge may indicate that the crystal field of 1T^′^-MoTe_2_ extends across the boundary into the 2H phase for two unit-cells. An analogous

phenomenon can be observed on the Te-M edge. The fine peaks on the Te-M edge ELNES from 570 eV to 630 eV arise from crystal field splitting of the Te-3d orbital. These peaks remain in the 1T^′^ phase near the boundary, but are broadened (Fig. 4e). At the boundary, the Te-M ELNES of 2H-MoTe_2_ also deviates from its intrinsic shape, indicated by the gray line in Fig. 4f. The atomic structure of the phase boundary deviates from the normal perfect lattice, resulting in reconstruction of the crystal field in a localized region. Other phase boundaries with different tilt angles exhibit similar behaviors in the Te-N and Te-M ELNES and similar two unit-cell interaction ranges, as shown in Figs S8 and S9 in the online supplementary data.

Broken translational symmetry at structural defects is often accompanied by changes in electronic structures. Previous studies reported that the boundaries in TMD materials could influence their optical and electronic properties [39–42], because of differences in the atomic arrangements between the boundaries and the bulk parent phase. In this work, we correlate the electronic structures with the atomic arrangements (tilt angle) and find that such microstructure (angle)-dependent behaviors are different for different physical excitation processes, that is the angle dependence is insensitive to the excitonic and inner shell excitations but sensitive to the plasmon oscillations. The energy loss peak of plasmon oscillations at interfaces between 1T^′^-MoTe_2_ and 2H-MoTe_2_ with large tilt angles shows substantial red-shift, which is expected to introduce high carrier injection barriers. This may be because of imperfect interfacial atomic arrangements. Such a strong angle dependence of plasmon oscillation indicates that adjusting the relative orientations of the two crystal grains of a heterophase structure provides a new strategy for controlling boundary electronic structures and further tuning contact properties. Therefore, in future, angle-controllable synthesis technologies may be used to make metal-semiconductor 2D heterostructures satisfy contact requirements in nanoelectronics. Moreover, as the band structure can be altered at boundaries [42,43], we also expect that phase boundary engineering with precisely designed tilt angle between two phases, would allow us to tune the local band structure and further manipulate the electrical and optical properties.

## CONCLUSION

In summary, we used monochromatic STEM-EELS with high spatial resolution and high energy resolution to study the atomic and electronic structures of 1T^′^/2H-MoTe_2_ phase boundaries with various tilt angles across a wide spectral range. The VEELS of 2H-MoTe_2_ incorporated five exciton peaks that extend through the boundary by 100–150 nm. The Te-N and Te-M core losses exhibited 1T^′^-MoTe_2_ features for a distance of two unit-cells in the 2H phase, indicating that the 1T^′^-MoTe_2_ crystal fields penetrated the boundary and extended a short distance into the 2H phase. Interestingly, the π+σ mode of DUV plasmon oscillations exhibited strong angle dependence. There is a red-shift of approximately 0.3–1.2 eV within a 4 nm area for large tilted phase boundaries, indicating change of dielectric function as well as the barrier for carrier injection. In contrast, no substantial shift is observed for near-zero and 60° tilted boundaries. Our atomic-scale measurements using STEM-EELS help to elucidate the properties of coplanar metal-semiconductor contacts in TMDs and shed light on electrical and photoelectrical device design via phase boundary engineering.

## Supplementary Material

nwaa087_Supplement_File
