# Supplementary material for: Correlating the electronic structures of metallic/semiconducting MoTe2 interface to its atomic structures
Source: Natl Sci Rev. 2020 Apr 29;8(2):nwaa087. doi: 10.1093/nsr/nwaa087 (PMC8288393; doi:10.1093/nsr/nwaa087)
Supplement: nwaa087_Supplement_File [file nwaa087_supplement_file.docx]

**Supplementary Data for：**

**Correlating the electronic structures of metallic/semiconducting MoTe_2_ interface to its atomic structures**

Bo Han^1,2^, Chen Yang^3,4^, Xiaolong Xu^3^, Yuehui Li^1^, Ruochen Shi^1^, Kaihui Liu^3^, Haicheng Wang^5*^, Yu Ye^3,6*^, Jing Lu^3,4,6^, Dapeng Yu^3,7^, and Peng Gao^1,6*^

1. Electron Microscopy Laboratory and International Center for Quantum Materials, School of Physics, Peking University, Beijing 100871, China
2. Department of Material Physics and Chemistry, University of Science and Technology Beijing, Beijing 100083, China
3. State Key Laboratory for Artificial Microstructure & Mesoscopic Physics, School of Physics, Peking University, Beijing 100871, China
4. Academy for Advanced Interdisciplinary Studies, Peking University, Beijing 100871, China
5. State Key Laboratory of Advanced Materials for Smart Sensing, GRINM Group Co. Ltd., Beijing 100088, and GRIMAT Engineering Institute Co. Ltd., Beijing 101402, China
6. Collaborative Innovation Center of Quantum Matter, Beijing 100871, China
7. Shenzhen Key Laboratory of Quantum Science and Engineering, Shenzhen 518055, China

***Corresponding authors.** E-mails:

hcwang@grinm.com;

[ye_yu@pku.edu.cn](mailto:ye_yu@pku.edu.cn);

[p-gao@pku.edu.cn](mailto:p-gao@pku.edu.cn)


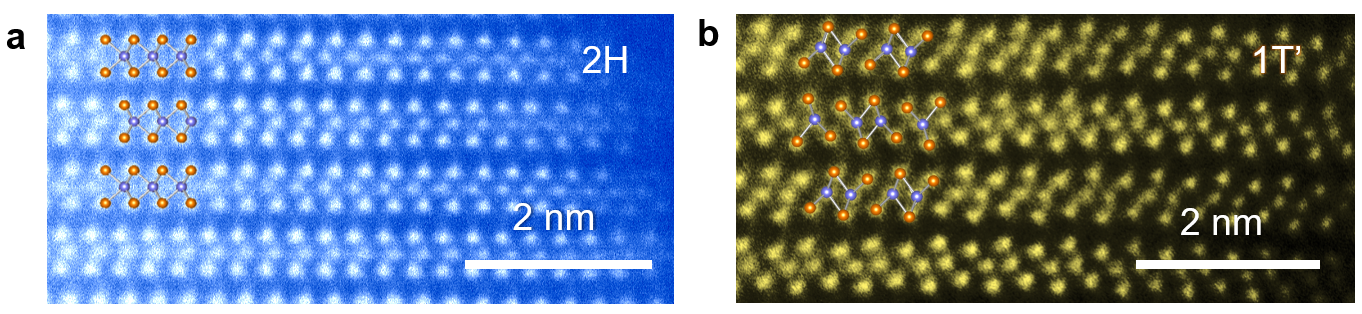


**Figure S1. The interlayer structures of (a) 2H-MoTe_2_ and (b) 1T’-MoTe_2_.** The corresponding atomic models are overlapped on HAADF images respectively.

From the interlayers structure of 1T’-MoTe_2_, the offset of Te-atom layers can be unambiguously observed, leading to a distorted octahedral arrangement round Mo atoms.


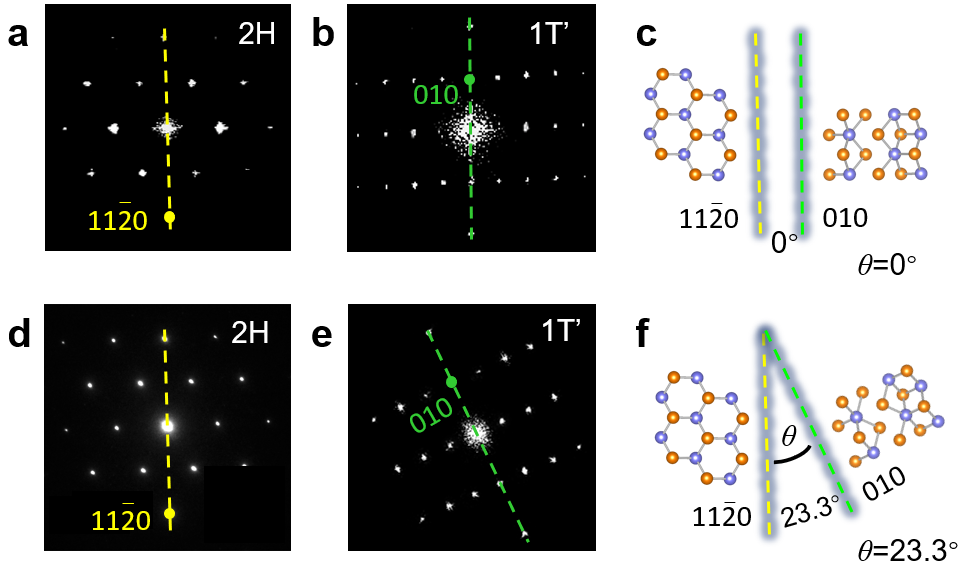


**Figure S2. Determination of tilt angles of MoTe_2_ phase boundaries.** A fast Fourier Transform (FFT) pattern of (a) 2H-MoTe_2_, and (b) 1T’-MoTe_2_ of the ~0° tilted boundary. (c) The sketch of the 0° tilted boundary. (d) The diffraction pattern of 2H-MoTe_2_, and (e) the FFT pattern of 1T’-MoTe_2_ in the ~23.3° tilted boundary. (f) The sketch of the 23.3° tilted boundary.

The tilt angle *θ* is the angle between the zigzag (<11$\bar{2}$0>) direction of the 2H phase (yellow dashed line) and the [010] direction of the 1T’ phase (green dashed line). These two directions are measured based on reciprocal patterns of 2H-MoTe_2_ and 1T’-MoTe_2_, as shown in Figure S2 a–b and Figure S2 d–e. The FFT patterns are produced using atomically resolved STEM-HAADF images.


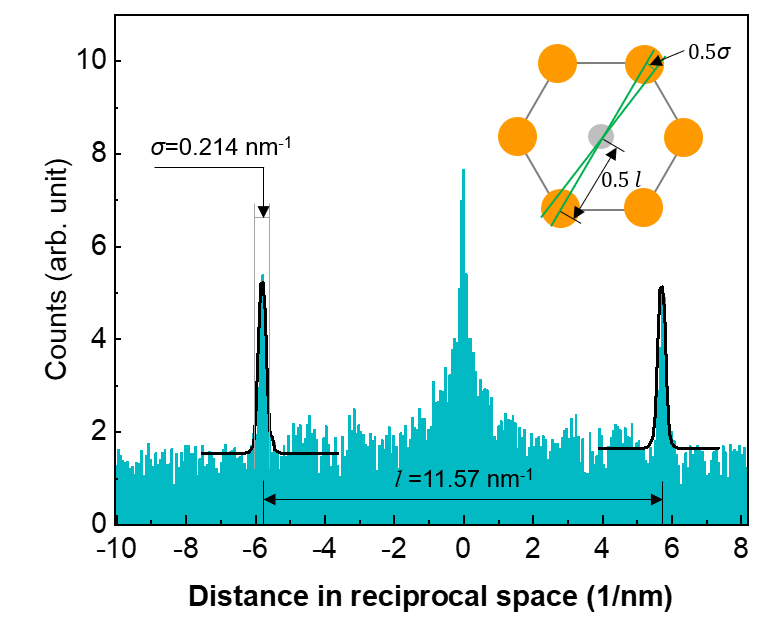


**Figure S3. Uncertainties of tilt angles measurements.** Orange circles are the first order diffraction FFT pattern of a 2H-MoTe_2_. Green lines indicate the line profiles position for tilt angle measurements. The cyan line profile shows the intensity distribution between a pair of (11$\bar{2}$0) FFT spots. The black profiles represent the Gaussian function fitted results of the spots.

In order to improve the measurement accuracy, a tilt angle is determined by FFT pattern of STEM images. The uncertainty mainly originates from the measurement uncertainty in reciprocal space, i.e., the position difference of profiling lines between two diffraction spots.

We fit each diffraction spot with a Gaussian function and its full width at half maximum (FWHM) is used to present the uncertainty.

The uncertainty of a tilt angle can be calculated by

$$\triangle\theta\approx arctan\left( \frac{\sigma}{l} \right)$$

where *σ* is FWHM of the Gaussian function for fitting a diffraction spot, *l* represents the distance between two symmetric FFT spots (not only the first order spots) of 2H or 1T’ MoTe_2_.

For instance, for a 2H-MoTe_2_, *σ* is measured to be 0.214 nm^-1^, *l* is measured to be 11.57 nm^-1^ between a pair of (11$\bar{2}$0) spots. The uncertainty of the tilt angle can be calculated by:

$$\triangle\theta\approx\arctan\left( \frac{\sigma}{l} \right)=\mathrm{arcta}n\left( \frac{0.214}{11.57} \right)=1.06^{\circ}$$

Note that because *σ* is << than L, we can use the above simplification of tan$\theta$=*σ*/*l*. Therefore, this method of measuring tilt angles in reciprocal space should be relatively accurate.


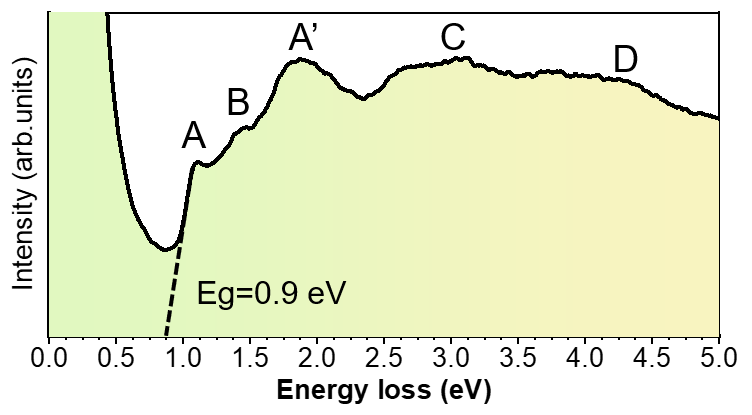


**Figure S4. The band gap measurement of 2H-MoTe_2_.**

The band gap of 2H-MoTe_2_ is 0.9 eV on basis of VEELS. The characteristic exciton (electron-hole pairs) peaks A and B represent the direct transition at K point in the Brillouin zone. The energy difference between peak A and B is of 300 meV due to spin orbit coupling, which is consistent with optical characterization. Peak A’ is caused by interlayer transition while peak C arises from the parallel bands at Γ point. [1]

It is worth mentioning that compare to the optical technique that is primarily sensitive to direct transition behavior, STEM-EELS has the advantages to mapping excitons at subwavelength scales [2] and probe the indirect transition of electrons and transition from deeper energy levels, which probably leads to the peak D at higher energy range (~4.3 eV).


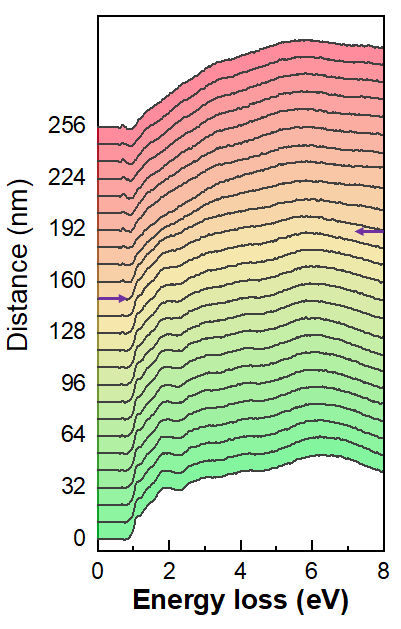


**Figure S5.** Valence EELS series across an ~21.9° tilted 1T’/2H-MoTe_2_ phase boundary.

The VEELS of an ~0° tilted 1T’/2H-MoTe_2_ phase boundary indicates that the interaction range is ~100–150 nm across the phase boundary, which also shows the same behavior as the boundaries with large tilt angles.


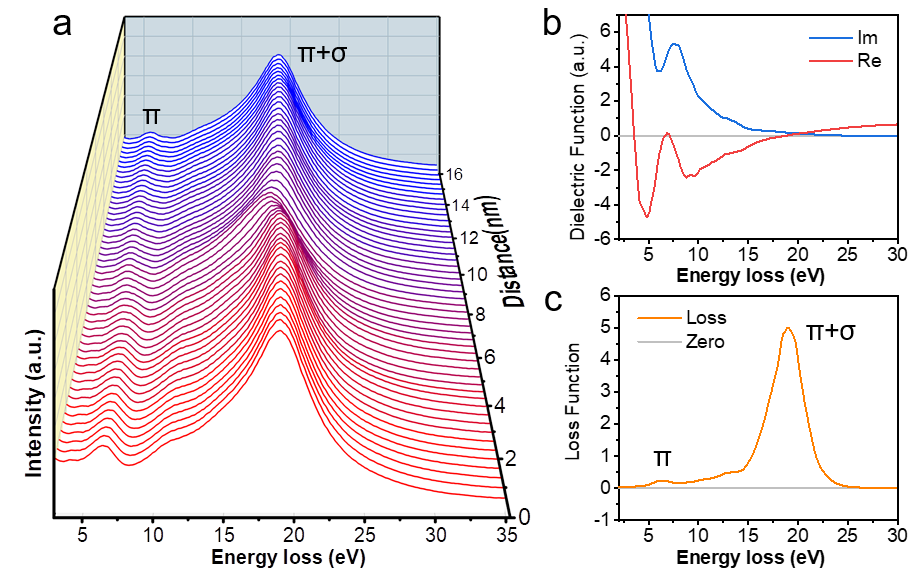


**Figure S6. The** **experimental EELS and calculated** **dielectric function of 2H-MoTe_2_.** (a) Spatial resolved plasmon oscillation of a 1T’/2H-MoTe_2_ boundary (tilted angle ~ 8.6°). The π energy loss peak MoTe_2_ locates at 6.4 eV (2H) and 5.7 eV (1T’). The energy loss position of π+σ peak is about 19 eV. (b) the dielectric function of 2H-MoTe_2._ (c) the calculated loss function based on dielectric function in (a).

The longitudinal wavelike oscillations of MoTe_2_ weakly bound electrons generate the plasmon and cause the energy loss.

The energy loss function *L* is calculated on the grounds of the function:

$$L=-I_{m}\left[ \frac{1}{\varepsilon\left( \omega\right)} \right]$$

where $\varepsilon\left( q,\omega\right)$ is the dielectric function produced by first principles calculations. The energy loss function of 2H-MoTe_2_ incorporates π (6.4 eV) and π+σ (19.1 eV) peaks, which are consistent with experimental observation.

Two dominated peaks π and π+σ can be observed in the energy loss range of 5–35 eV. The π plasmon mode in MoTe_2_ results from the π-π* transitions, while π+σ plasmon mode arises from the π-σ* and σ-σ* excitations. [3]


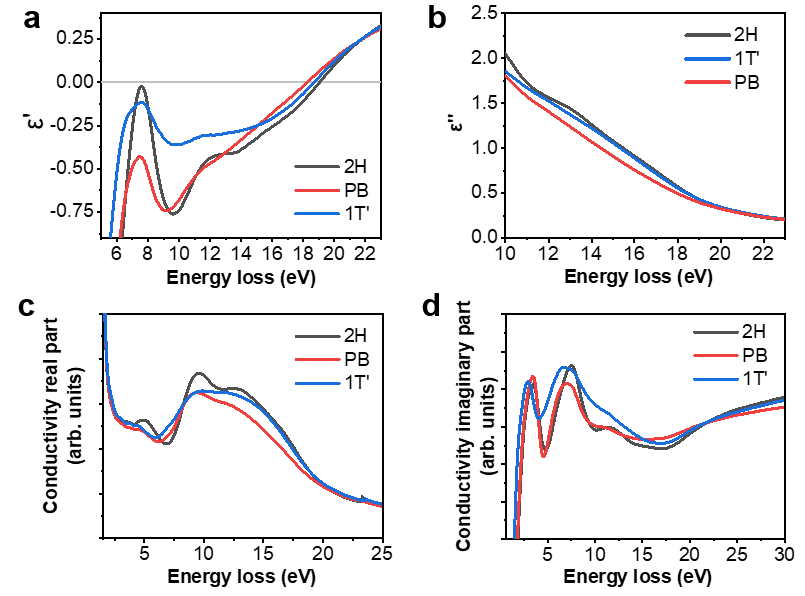


**Figure S7. The K-K analysis of plasmon mode of 1T’/2H-MoTe_2_ phase boundary.** (a) The real part and (b) the imaginary part of the dielectric function of 2H-MoTe_2_, 1T’-MoTe_2_, and the phase boundary (PB) are indicated by black, blue and red, respectively. (c) The real part and (d) the imaginary part of conductivity deduced from the dielectric functions.

The increased real part and the decreased imaginary part of the dielectric function at phase boundary (in the range of 15–21 eV) contributes to the energy red shift and intensity decrease of π+σ peak. The conductivity at the phase boundary slightly decreased compared to the intrinsic MoTe_2_.

The red shift of π+σ plasmon mode at the phase boundary is attributed to the reduction of effective electron density at the phase boundary. The free electron density valley at the phase boundary might generate a barrier for the carrier injection from the 1T’-MoTe_2_ to 2H-MoTe_2_, which is detrimental for contact.


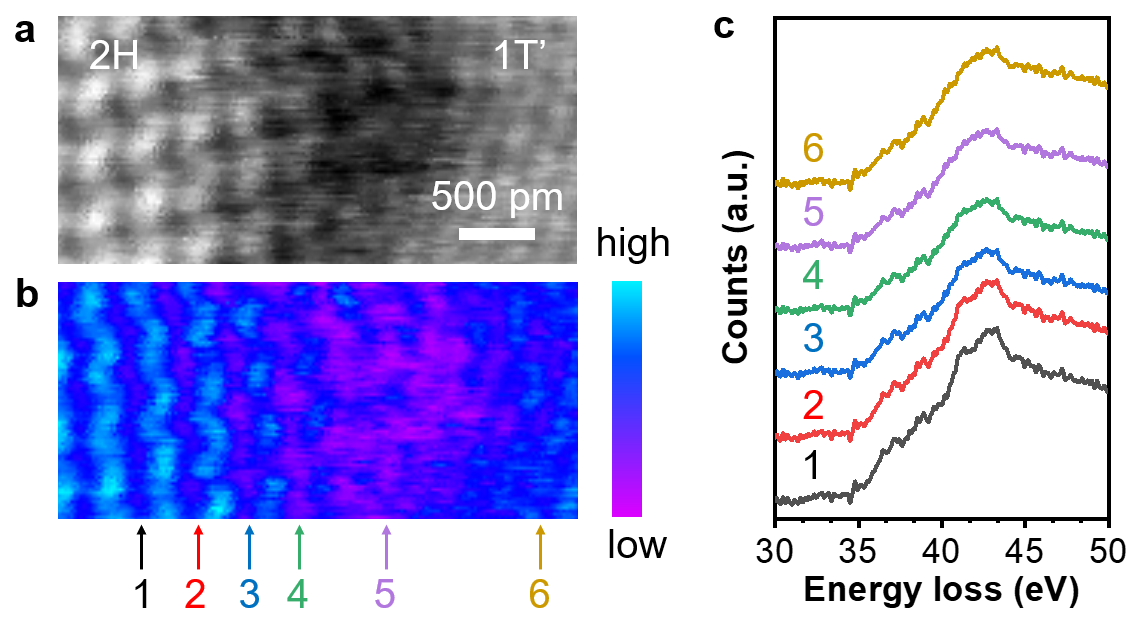


**Figure S8. Te-N ELNES at the GB of ~8.6° tilt angle.** (a) A HAADF image showing the spectra-collected region. (b) Spectral image of the Te-N edge. The spectral image is extracted by applying an integral window of 30-50 eV. (c) Te-N edges of six locations are indicated by black, red, blue, green, purple and yellow arrows in (b), respectively.

In this case, the fine structures of Te-N edge of 2H-MoTe_2_ also change within 2 unit cells at the ~8.6° tilted boundary, in a similar manner with the ~0° tilted boundary.

It is worth mentioning that the typical probe size is ~1.07 Å which can be measured based on the resolution of atomically resolved spectral image. As a result, the sampling region of atomically resolved STEM-EELS can be very local.


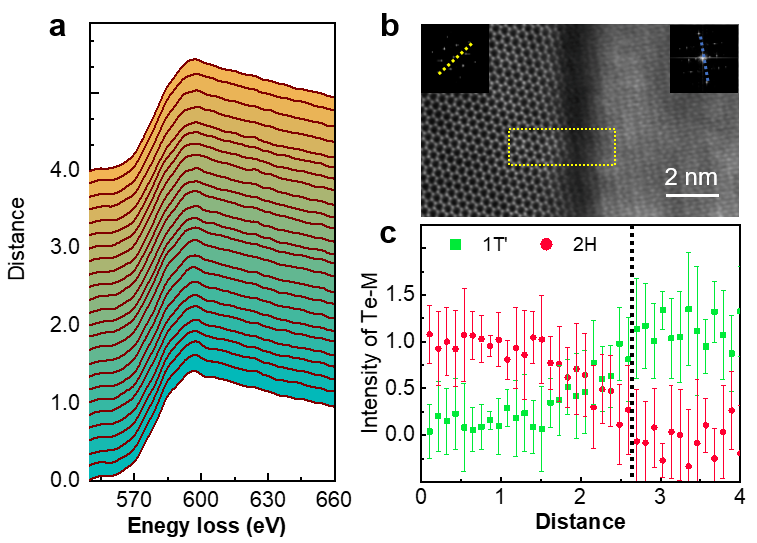


**Figure S9. Te-M** **ELNES at GB of 53.5° tilt angle.** (a) Spectra series across the 1T’/2H phase boundary. (b) A HAADF image showing the ~53.5° tilted phase boundary. (c) The Te-M intensity of 1T’ and 2H components plotted as a function of distance across the boundary. Red sphere: 2H; Green square: 1T’.

**METHODS**

**Synthesis of the MoTe_2_ Films.** The MoTe_2_ films were synthesized by tellurizing the Mo film at atmospheric pressure using a horizontal hot-wall tube furnace equipped with mass flow controllers and a vacuum pump. Mo films were deposited on Si/SiO_2_ substrates through magnetron sputtering. The substrates were placed face-down on an alumina boat containing Te powder placed at the center of the heating zone in a one-inch quartz tube. After evacuating the quartz tube to less than 1 mTorr, we flowed Ar gas at 500 standard cubic centimeters per minute (sccm) until the pressure reached atmospheric pressure. At atmospheric pressure, Ar and H_2_ flowed at rates of 4 and 5 sccm, respectively. The furnace was ramped to 650 °C in 15 min and was kept at the temperatures for 30 min to synthesize the coplanar contact structure. After the reactions, we let the furnace cool to room temperature naturally.

**Transfer of the 1T′/2H hetero-phase MoTe_2_ thin film.** First, drop a few mL of isopropanol (IPA) onto the hetero-phase MoTe_2_ thin film on the Si/SiO_2_ substrate. A mesh copper grid was carefully put onto the IPA solution. After the IPA evaporates naturally, the copper grid is glued to the hetero-phase MoTe_2_ thin film. Then, a small amount of the dilute HF solution (1.5%) was dropped onto the edge of the copper grid. A few seconds later, the copper grid along with the MoTe_2_ film floats in solution. Finally, the sample was thoroughly rinsed with DI water.

**STEM and EELS characterization.** The STEM-HAADF images were acquired using a Nion U-HERMES 200 microscope with both monochromator and the aberration corrector operating at 60 kV at room temperature. The convergence semi-angle was 35 mrad and the collection semi-angle was in the range of 80–210 mrad. In order to achieve high spatial resolution, the EEL spectrum images were recorded at STEM mode with a 1 mm spectrometer entrance aperture. The STEM-EELS were recorded at 60 kV with a collection semi-angle of 24.9 mrad. The EELS background was fitted and subtracted using power law $I\left( \Delta E \right)=A_{0}\cdot{\Delta E}^{-r}$. The interfacial VEELS were fitted by multiple linear least-squares (MLLS) method using DigitalMicrograph (Gatan) software. The intrinsic VEELS of 1T’-MoTe_2_ and 2H-MoTe_2_ and were used as reference spectra respectively. The spectra were normalized using the zero-loss peak of EELS. The low loss plasmon EELS spectrum for K-K analysis was Fourier-log deconvolved in order to extract the single scattering distribution [4].

**DFT calculation.** The geometry optimizations of both 1T’ and 2H-MoTe_2_ have been fully calculated before the electronic structure calculations. The plane-wave basis set with a cut-off energy of 400 eV and the projector augmented wave (PAW) pseudopotential are used in the Vienna *ab initio* simulation package (VASP). Throughout the calculations, the generalized gradient approximation (GGA) combined with the Perdew-Burke-Ernzerhof (PBE) form are adopted. The maximum residual force of per atom is less than 0.001 eV/Å to obtain a reliable optimized structure, and the convergence standard of energy on each atom is within 1 × 10^-6^ eV. We use the Monkhorst–Pack method to sample the *k*-point mesh with a separation of 0.04 Å^-1^ and 0.02 Å^-1^ in the irreducible Brillouin zone (IBZ) for the geometry optimizations and electronic structure calculations, respectively.

**REFERENCES**

1. Ruppert C, Aslan OB andHeinz TF. Optical properties and band gap of single- and few-layer MoTe_2_ crystals. Nano Lett 2014; **14**: 6231-6.

2. Tizei LH, Lin YC, Mukai M *et al.* Exciton mapping at subwavelength scales in two-dimensional materials. Phys Rev Lett 2015; **114**: 107601.

3. Liang WY andCundy SL. Electron energy loss studies of the transition metal dichalcogenides. Philosophical Magazine 1969; **19**: 1031-1043.

4. Egerton RF. Electron energy-loss spectroscopy in the electron microscope[M]. 3nd. Springer Science & Business Media, 2011.
